# Artificial intelligence to predict soil temperatures by development of novel model

**DOI:** 10.1038/s41598-024-60549-x

**Published:** 2024-04-30

**Authors:** Lakindu Mampitiya, Kenjabek Rozumbetov, Namal Rathnayake, Valery Erkudov, Adilbay Esimbetov, Shanika Arachchi, Komali Kantamaneni, Yukinobu Hoshino, Upaka Rathnayake

**Affiliations:** 1 Water Resources Management and Soft Computing Research Laboratory, Athurugiriya, Millennium City, 10150 Sri Lanka; 2 Department of Anatomy, Physiology and Biochemistry of Animals, Nukus Branch of Samarkand State University of Veterinary Medicine, Animal Husbandry and Biotechnology, 230100 Nukus, Uzbekistan; 3 Department of Civil Engineering, Faculty of Engineering, The University of Tokyo, 1 Chome-1-1 Yayoi, Bunkyo City, Tokyo, 113-8656 Japan; 4 Department of Normal Physiology, St. Petersburg State Pediatric Medical University, 194100 Saint Petersburg, Russia; 5 Department of Electronics and Mechanical Engineering, Faculty of Engineering and Technology, Atlantic Technological University, Letterkenny, F92 FC93 Ireland; 6 UN-SPIDER-UK Regional Support Office, University of Central Lancashire, Preston, PR1 2HE UK; 7 School of Engineering, University of Central Lancashire, Preston, PR1 2HE UK; 8 School of Systems Engineering, Kochi University of Technology, 185 Miyanokuchi, Tosayamada, Kami, Kochi 782-8502 Japan; 9 Department of Civil Engineering and Construction, Faculty of Engineering and Design, Atlantic Technological University, Sligo, F91 YW50 Ireland

**Keywords:** Artificial intelligence, Climatic parameters, Machine learning, Prediction, Soil temperature, Uzbekistan, Environmental impact, Solid Earth sciences

## Abstract

Soil temperatures at both surface and various depths are important in changing environments to understand the biological, chemical, and physical properties of soil. This is essential in reaching food sustainability. However, most of the developing regions across the globe face difficulty in establishing solid data measurements and records due to poor instrumentation and many other unavoidable reasons such as natural disasters like droughts, floods, and cyclones. Therefore, an accurate prediction model would fix these difficulties. Uzbekistan is one of the countries that is concerned about climate change due to its arid climate. Therefore, for the first time, this research presents an integrated model to predict soil temperature levels at the surface and 10 cm depth based on climatic factors in Nukus, Uzbekistan. Eight machine learning models were trained in order to understand the best-performing model based on widely used performance indicators. Long Short-Term Memory (LSTM) model performed in accurate predictions of soil temperature levels at 10 cm depth. More importantly, the models developed here can predict temperature levels at 10 cm depth with the measured climatic data and predicted surface soil temperature levels. The model can predict soil temperature at 10 cm depth without any ground soil temperature measurements. The developed model can be effectively used in planning applications in reaching sustainability in food production in arid areas like Nukus, Uzbekistan.

## Introduction

Soil is a three-phase system consisting of solids, water, and air. The long-term balance of physical components of soil is highly essential not only to humans but also to all other living things of the world^1^. Therefore, it is important to understand the anthropogenic activities that directly and indirectly impact the balance of the soil system. Development activities have significantly contributed to the unbalance of the soil systems^2^. In addition, climate change is one of the main contributors to unbalancing the soil system^3^. Climate change has significantly impacted the soil system in various ways. This is due to dramatic changes in rainfall patterns, atmospheric temperature, and other climatic conditions^4,5^. Significant attention was/is given to the impact of climate change on various clusters of nature including water resources^6^, energy harvesting^7^, food and agriculture^8^, etc. However, understanding soil temperature changes over time is not given a comprehensive understanding in most of the developing regions where directly involved with agriculture. Nevertheless, soil temperature is one of the main factors that is capable of impacting plants and other microbial activities in the soil. Maintaining the proper soil temperature, therefore, is an important task, to control the balance of nature. Sudden changes in the soil temperature significantly impact the sensitive crops that are grown only in a specific season of the year^9^. Therefore, the food production is critically impacted^10^.

Because of those factors, directly and indirectly, the soil temperature affects the lifestyles of all the living beings on Earth. Such critical cases could cause the diversity changes of the animals^11^. The damage that occurs to their habitats is one of the major concerns^12^. Seasonal variations which naturally occur are adapted by the other stakeholders of the environment^12^. However, sudden and abrupt changes are to be watchful. Therefore, proper understanding of soil temperatures is important. In addition, accurate prediction of soil temperatures under the changing climate is equally important. The sudden higher and lower temperatures affect the biological components in the soil^13,14^. The higher soil temperatures lead to an increase in the respiration rate of the ecosystem^15,16^. Therefore, the overall carbon balance in the ecosystem could be subjected to variations^17^. On the other hand, low soil temperatures could affect the plant growth in many ways. The plant growth can be reduced due to the limitations applied to the root cause expansion and the photosynthesis process of the plants^18,19^. Topsoil temperature levels (0–1 m) are important for most of the agricultural activities^20^. However, these temperature levels are not monitored by most of the developing countries. In addition, some of the existing monitoring stations are not properly maintained^21^, therefore, the soil temperature recordings along the depth are minimal. However, understanding the temperature variation for the first 10 cm layer would help the researchers to project the adverse impact^22^. This understanding has not been done to most of the developing countries including Uzbekistan where they rely on agriculture.

Uzbekistan is located in Central Asia with an arid climate due to its geographical conditions which include several deserts^23^. Water shortage, soil salinity, and adverse climatic conditions are rising environmental problems in the country^24^. Soil salinity combined with water shortage and changing soil temperatures worsens the agricultural production in the country^25,26^. Indirectly, the quality of life is significantly degraded. Therefore, it is visible that consideration of the soil condition is really important for a country like Uzbekistan. Soil temperature is one of the main factors that lead to the soil salinization process as it directly contributes to the evaporation of the water content in the soil^4^. On the other hand, soil salinization is one of the factors that contribute to the mineralization process of the soil^27^. Different climate factors contribute to accelerating the processes of microbes. Affecting the speed of microbial activities would lead to the speed variation of the decomposition process of the soil organic materials. These would worsen the soil situation in Uzbekistan. Considering the above factors, it is visible that soil temperature controls the many factors that are connected with the environment directly and indirectly. On the other hand, this could lead to economic-level changes and changes in the quality of life^6^. Therefore, it is evident that countries like Uzbekistan need an accurate system to predict soil temperatures. The main advantages of this are, that the fertility of the soil can be controlled and maintained at a satisfactory level, the water content of the soil can be controlled, and the functioning of the microbial activities in the process of decomposing the soil organic materials. Additionally, indirectly then this supports economic growth and the quality of life.

Therefore, this research presents the first-ever study that predicts the soil temperature at under external environmental factors like climatic factors in one of the important areas of Uzbekistan, Nukus. Soil surface temperatures and 10 cm depth soil temperatures are successfully predicted under the changing environmental conditions. The research presented herein has a greater novelty as this is the first-ever study carried out in the context of predicting soil temperatures to Nukus with the usage of machine learning techniques. Soil temperature at 10 cm depth is highly important for the initial stage of cultivation including seed germination. The moisture level of the topsoil is highly important to the initial stages of cultivation. Therefore, the 10 cm depth was given higher attention. In addition, the data for the total root zone were not available for the area. Therefore, a comprehensive understanding of soil temperature variation throughout the soil profile in the root zone is unavailable.

## Study area and dataset

**Figure 1:**
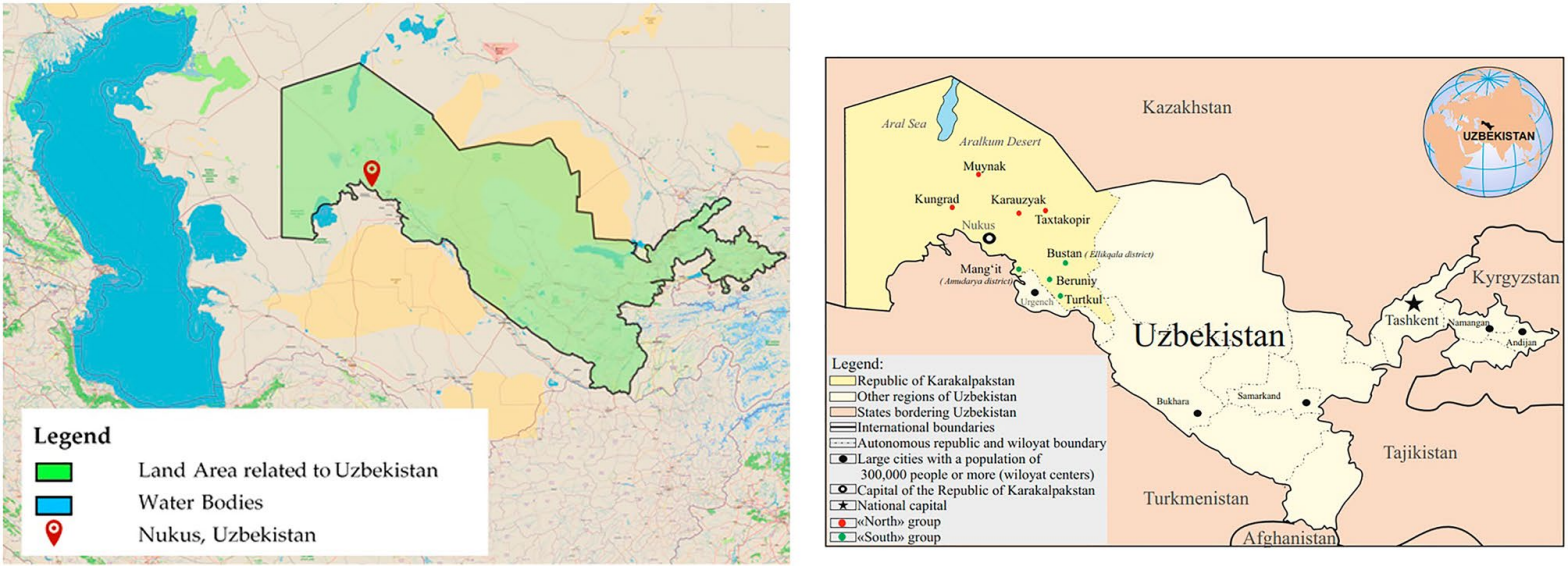
Study area—Nukus, Uzbekistan (software used—QGIS version 3.36.1 & Microsoft Paint version Office 365).

This research study considers Nukus, Uzbekistan is the capital of the sovereign Republic of Karakalpakstan within Uzbekistan and it is the sixth largest city in Uzbekistan. It is a traditional agricultural area located in northwestern of Uzbekistan. Cotton and grain are major agricultural products of the region^28^. The area experiences cold desert climate with high summer temperatures. Nevertheless, it is an area for 332,500 people (as per the census in 2022)^28,29^. Figure 1 illustrates location of Nukus (42.4619° N, 59.6166° E). Moreover, it shows the water bodies, vegetation, and some details about the land area (deserts). The map was developed using the freely available software modules QGIS version 3.36.1 and Microsoft Paint version office 365.

### Types of soil in Uzbekistan and variations in soil

Different types of soil compositions can be observed in Uzbekistan^30^. The distribution of the soil types over the county can be identified as follows in Table 1.

**Table 1:** The soil type and the distribution percentage.

Types of soil	Land area covered (%)
1. Grey-brown desert	25
2. Sandy	3
3. Takyr	4
4. Meadow-takyr	1
5. Meadow and wetland-meadow	4
6. Solonchak	3
7. Sands	28
8. Light serozem	6
9. Typical zerozem	7
10. Dark serozem	2
11. Brown and brownish middle altitude	4
12. Light brownish high-altitude	1
13. Meadow-serozem	2
14. Meadow and wetland-meadow	2
15. Rock	6

Source^30^.

According to the statistics represented, most of the land area of Uzbekistan is covered with the Grey-brown desert, 28%. Therefore, water scarcity and then limitations of agriculture can be often observed. However, Nukus, where this research study is spotted is composed of the Gray Desert Soil. Lesser organic materials with high salinity levels can be seen in Nukus.

### Ecosystem and environmental conditions

Uzbekistan has a unique ecosystem that consists of different bio-diversity patterns. According to the United Nations Development Program, it was proven that 80% of the water in Uzbekistan originated in neighboring countries^31^. However, water resources are limited in the country. Soil erosions, soil degradations, and water quality issues are some other related environmental issues that can be seen in the region.

### Soil temperature and climatic contribution

Climatic factors have a significant relationship to the soil temperature in the considered area, Nukus^32^. Therefore, three climatic factors including atmospheric temperature, relative humidity, and wind speed were observed in this research. Nukus has the highest atmospheric temperature in July (can be higher than 35 °C) but the lowest in January (can be lower than − 2.7 °C). However, Nukus receives around 100 mm of annual rainfall, thus the variation is insignificant for monthly rainfalls.

### Geographical factors contribution to soil temperature

Geographical features of the Nukus are important and contribute to soil temperature. Nukus is located at an altitude of 80 m. As it was stated, Uzbekistan receives most of the surface water from neighboring countries^31^. However, the Nukus area has some water bodies in the vicinity and listed below.

- a. Amu Darya River, there are branches of this river that flow through the Nukus. (This supports the fertility and maintenance of the Nukus soil temperature)
- b. Sarykamysh Lake, a water body that is situated at a distance of around 170 km
- c. kvadrat Lake, a minor lake that is situated at a distance of 4 km
- d. Kos Kul’ Lake, situated at a distance of 5 km

In addition, Karakum and Kyzylkum deserts are the nearest considerable areas. Therefore, the distances between deserts and Nukus, and the distances to the water bodies directly and indirectly impact the soil temperatures in Nukus. Furthermore, nearest mountain ranges are another important phenomenon that contributes to the soil temperature variations. The nearest mountain that is situated close to Nukus is Beshtobe Tog’. It is ~ 25 km away from the Nukus.

Therefore, it can be clearly seen that the soil temperature prediction is very important to the Nukus, Uzbekistan for future planning and then to reach sustainable development goals (SDG2, 3, 8 & 13).

### Dataset

Atmospheric temperature (AT) (minimum and maximum in °C), relative humidity (RH) (in %), and wind speed (WS) (in m/s) were considered herein to predict the soil temperature levels of Nukus. Therefore, monthly climatic data for Nukus from 1989 to 2018 (30 years) were obtained from Hydrometeorological Center of the Republic of Karakalpakstan. Three recordings were done for each month. Therefore, 1080 (30 × 12 × 3) datasets were obtained. Simultaneously, soil surface temperatures and 10 cm below the surface temperatures were obtained from the Hydrometeorological Center of the Republic of Karakalpakstan. In addition, to understand the temporal variation, the rainfall data were also collected.

### Machine learning techniques

At present, artificial intelligence has become one of the most utilized techniques in the world^33,34^. It has the capability of identifying unseen patterns, and complex interconnections in the data series. Machine learning models are capable of predicting nonlinear relationships with high accuracy^35,36^. Therefore, several (8) state-of-the-art machine learning models were utilized to predict the surface and 10 cm depth soil temperature.

- Extreme Gradient Boost Algorithm (XGBoost) is one of the commonly used algorithms in artificial intelligence for predictions. The model tries to minimize the error through each step by following the decision tree structure. XGBoost is capable of controlling its error^37^.
- Categorical Boost Algorithm (CatBoost)—CatBoost follows the same approach used in the XGBoost and it is a very powerful regression model. The main specialty of CatBoost is the efficient functioning of larger datasets and understanding the data patterns. Feature importance and parallel functioning are two main advantages of this model which leads to higher results.
- Long Short-Term Memory (LSTM)—LSTM almost follows the same structure of the RNNs to work on the regression. Handling complex data patterns and factor relationships are two of the important functionalities of the model. LSTMs overcome limitations like exploding and vanishing gradients, enabling them to effectively handle long-term dependencies in sequences. This highlights the advantages LSTMs offer compared to other models. The structure-wise, this is composed of the three main gate structures following the Input Gate, Output Gate, and Forget Gate^38,39^.
- Bi-Directional LSTM (Bi—LSTM)—RNN structure is capable of understanding more data patterns than the uni-directional LSTM. Functioning and understanding the noises of the datasets and handling them is one of the specific features of this model. Forward and backward passing functionality allows the models to minimize the error. Processing the sequence in both directions doubles the number of gates and weights compared to unidirectional LSTMs, leading to higher training time and resource requirements^40^.
- Artificial Neural Network (ANN)—ANN shows a higher possibility of understanding the linear and nonlinear combinations between the input factors and the output factors^39^. Moreover, due to the higher controllability of the models with the hyperparameters, the ANN models can be adapted to specific scenarios. Fine-tuning of the models leads to higher functionality of the model^41^.
- Ridge Regression—It is a state-of-the-art model that is developed to function over a case of overfitting and multicollinearity of the scenario. This approach modifies Ordinary Least Squares (OLS) regression by introducing an L2 regularization term proportional to the squared L2 norm of the coefficients. This is particularly advantageous in scenarios with high multicollinearity among independent variables, as it mitigates the issue of coefficient inflation and instability^42,43^.
- ElasticNet—One of the important factors about the ElasticNet is, that this is built upon the weak points of the Lasso and Ridge Regression. ElasticNet learns from the Lasso and Ridge regression shortcakes and improves the regularization of the model. In general, feature selection, robustness and better performance over a large number of dataset variables make this state-of-the-art model more compatible with this research study^43,43^.
- Least Absolute Shrinkage and Selection Operator (Lasso) Regression—This can be represented as the combination of the statistical models and the machine learning models. This allows the model to understand the relationships and the data patterns and to carry out predictions. Due to the controllability of the model, this can be fine-tuned for the specific case and make predictions more accurate. While the Ridge regression uses L2 Normalization, Lasso uses the L1 Normalization technique^43^.

The literature on machine learning approaches showcases the promising findings on environmental engineering applications^38,41,44,45^. The research works carried out by Mampitiya et al.^38^, showcased the applicability of AI in related environmental engineering problems.

AI is widely used for the prediction of soil temperatures. Mainly that’s because of the possibility of them understanding the data pattern variations. LSTMs and Gradient Boost algorithms were well utilized for these scenarios^46^. Applying the state-of-the-art model undergoes several steps when it comes to real-world applications. Ozturk et al.^47^ illustrated the usage of the Artificial Neural Network in the prediction of soil temperature. Moreover, they have represented how the prediction moves on with the different depth levels. Even though the state-of-the-art model performance is more promising, the frequency of data catching plays a crucial role in that.

## Methodology

**Figure 2:**
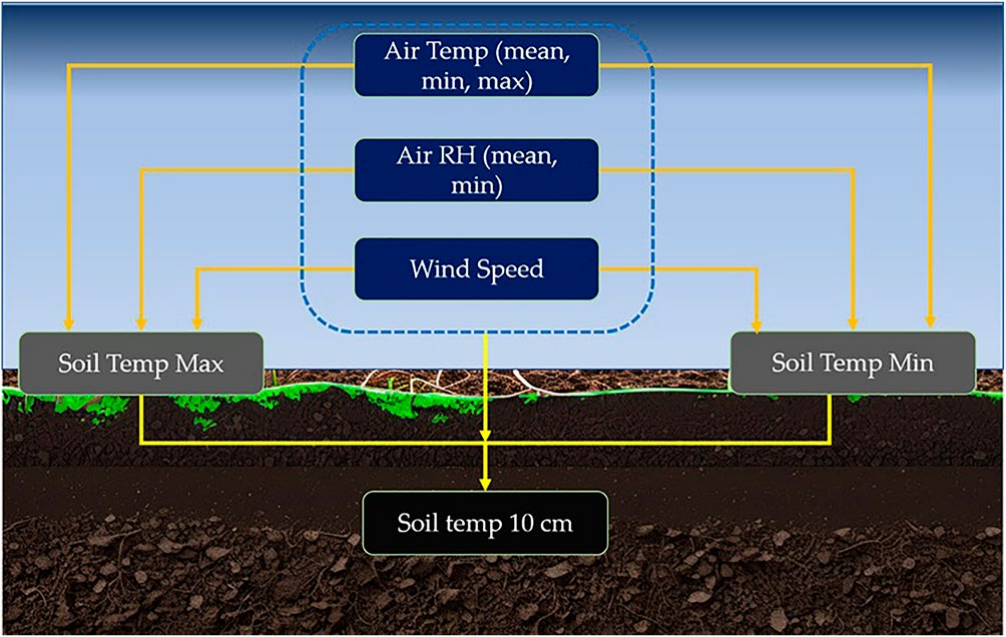
Different factor contribution for the surface and 10 cm depth soil temperature.

The main objective of this research is to predict the soil temperature levels at soil surface and 10 cm below the surface based on climatic parameters. Atmospheric temperatures (air temperatures), relative humidities, and wind speeds were considered the external climatic parameters for the model development. Figure 2 showcases the factors contributed to the surface soil temperature and the 10 cm depth soil temperature. These data for the 30 years from 1989 to 2018 were obtained.

### Data cleaning

Data cleaning is a crucial step in processing the data for machine learning models. Removal of the noises of the data and the handling of the missing values were two important cases in data cleaning. The unacceptable data variations were removed with the support of field expertise. One of the major issues that arises during the data handling is, missing data of the dataset. These dataset factors are interconnected in the view of environmental engineering; thus, the missing values in the dataset may lead to a break in the data pattern. Those missing data were also removed to bring a solid dataset for the assessment.

### Prediction functions development

This research work was carried out in two steps, where the prediction of the surface soil temperature was carried out as the first step and the prediction of the 10 cm depth soil temperature based on the surface temperature was the second step. Equation (1) showcases the mathematical formulation to predict the minimum and maximum soil temperature and surface.

1 $${T}_{surface}=Function({AT}_{Mean},{AT}_{Max},{AT}_{Min},RH,WS)$$

Equation (2) presents the mathematical formulation to predict the soil temperature at 10 cm below the surface.

2 $${T}_{10cm}=Function({AT}_{Mean},{AT}_{Max},{AT}_{Min},RH,WS,{T}_{surface,min},{T}_{surface,max})$$

### Machine learning models development

**Figure 3:**
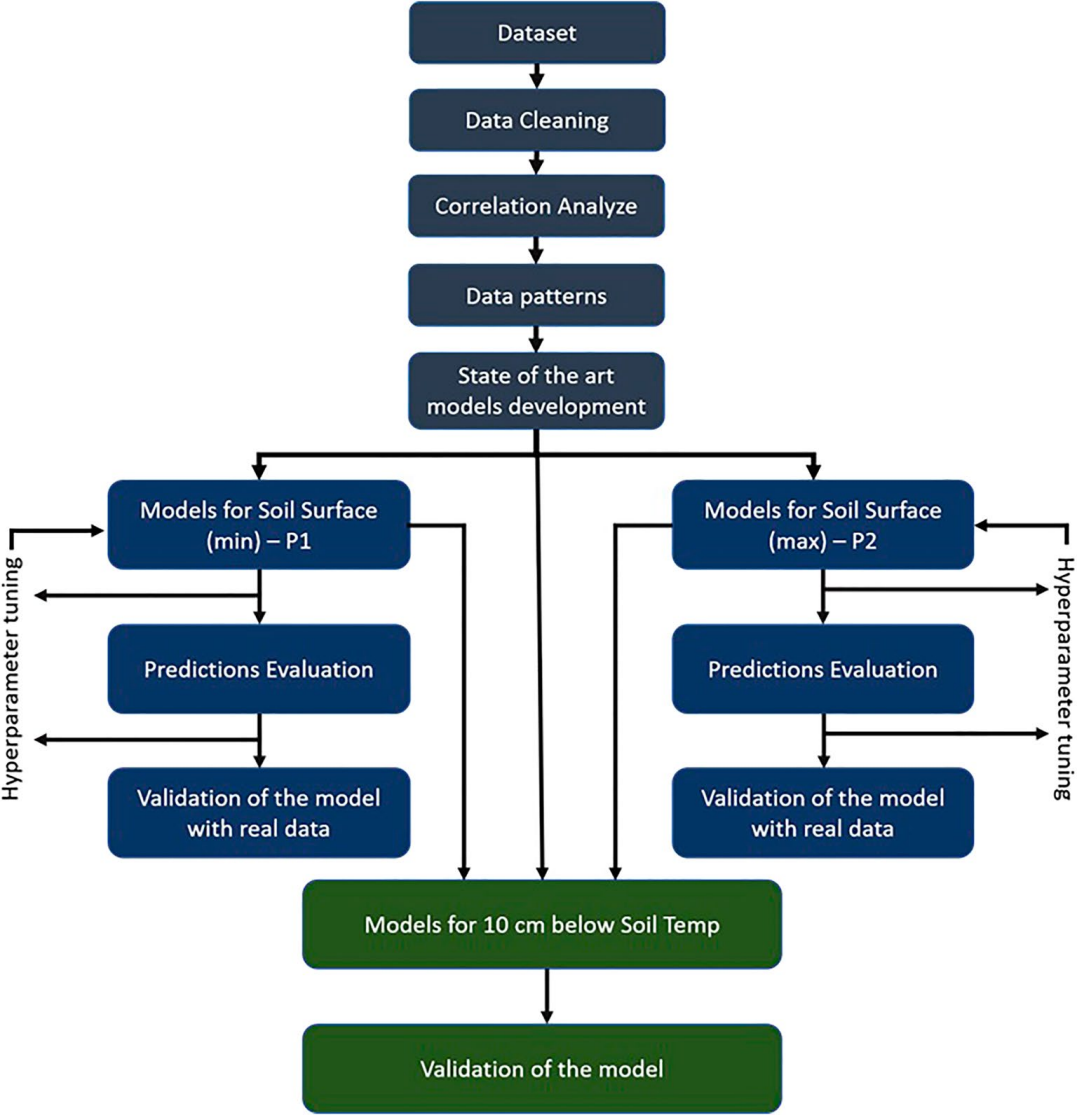
Overall methodology.

Eight state-of-the-art machine learning models including XGBoost, CatBoost, LSTM, ANN, Bi-LSTM, Ridge Regression, Lasso Regression, and ElasticNet were utilized in formulating the two equations (Eqs. 1, 2). Figure 3 presents the overall methodology which was carried out to predict the soil temperatures.

Overfitting is one of the main concerns in many machine-learning approaches. Therefore, regularization techniques, hyperparameter tuning with the evaluations, and k-fold cross-validation techniques were utilized to minimize the overfitting. In addition, early stopping of the models was carried out in this study to avoid overfitting. Moreover, the complexity of the algorithms was tuned to achieve maximum performance by avoiding the overfitting of the models.

### Performance evaluation of the models

Under this step, the whole functionality of the models was examined. To gain the justification of the results, four evaluation matrices were concerned over the models and analysed to identify the best-performing model over the scenario. Regression coefficient (${R}^{2}$), Root Mean Square Error (RMSE), Mean Absolute Error (MAE), and Mean Square Error (MSE) were used in the evaluating matrices.

## Results and discussion

### Temporal variation of soil temperature

**Figure 4:**
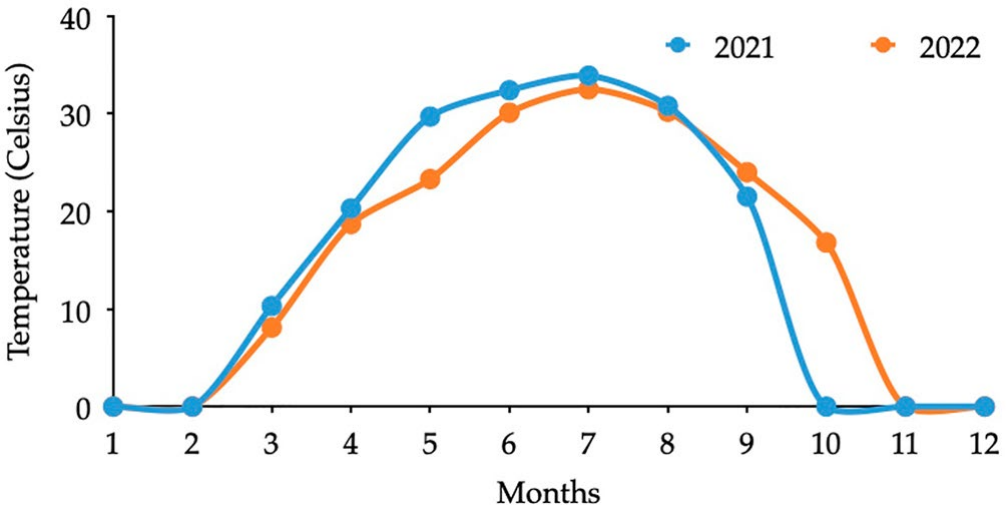
10 cm depth soil temperature variation.

The interdependency of the involved factors was identified. It is usual to observe increasing temperatures with the depth of soil. However, a different variation was observed in the dataset from the surface to the 10 cm depth. Soil temperature seems to be lower than the surface and this could be due to the water content and the minimal contact with solar radiation. In addition, a clear seasonal variation can be found in soil temperatures at 10 cm. Figure 4 presents the soil temperature variation at 10 cm for a couple of years. Higher soil temperatures can be observed during the summer months of June–September but lower temperatures during the winter months.

### Temporal variation of soil temperature with other variables

**Figure 5:**
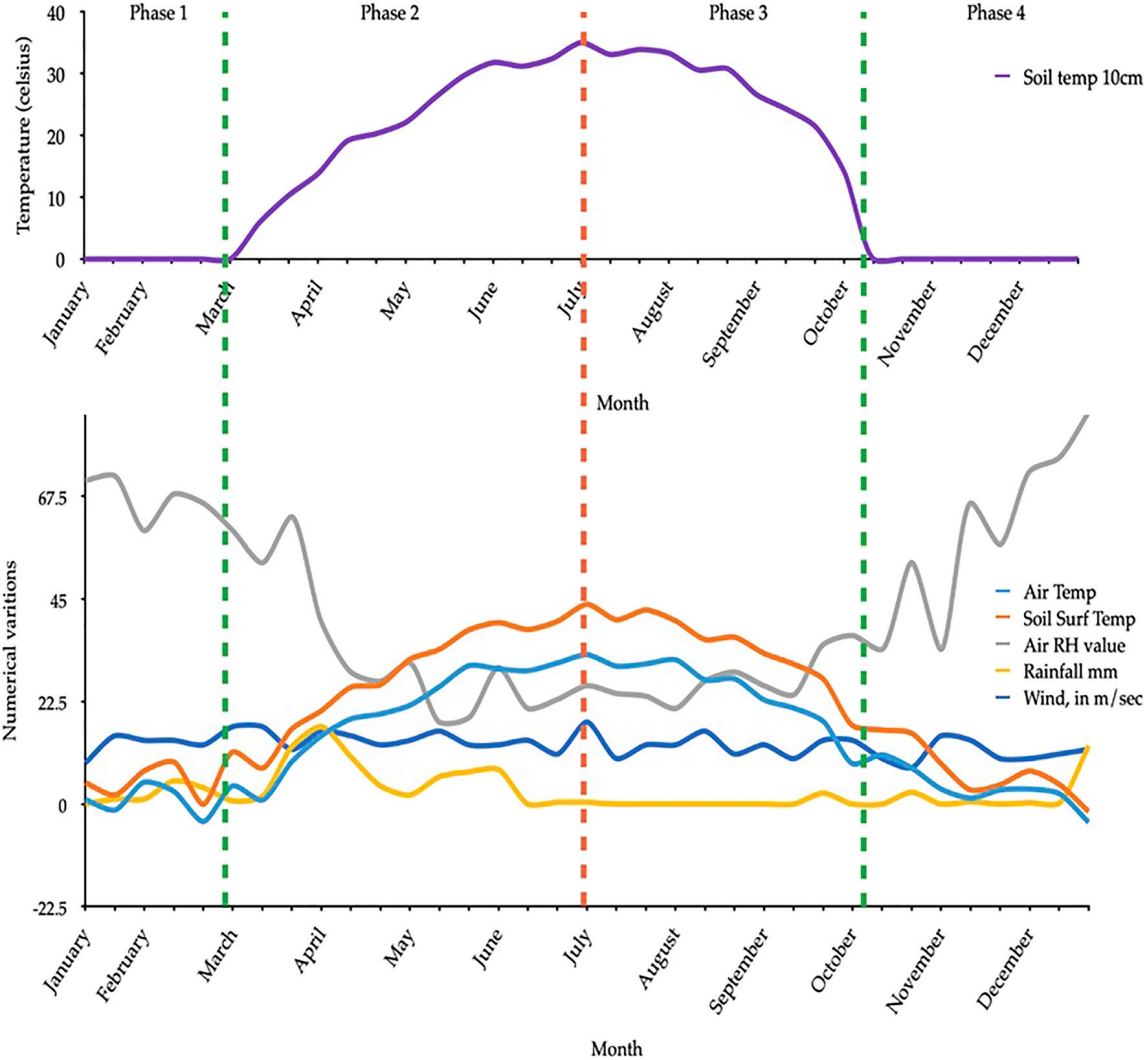
Temporal variation of soil temperature at 10 cm depth.

Figure 5 presents the soil temperature at 10 cm depth against the other variables along the time. Four phases can be identified to discuss the temporal variation and the connection among the variables. Phase 1 illustrates how the variations change between January and March. It represents the period where the 10 cm depth temperature is around 0 °C. During this period, the other factors represent negative and positive rises. Air relative humidity represents a considerable variation. Under phase 2, the rising edge of the 10 cm depth temperature can be observed. The soil surface temperature rises, and a sudden drop in relative humidity can be seen. The soil temperature at 10 cm depth decreases in phase 3. At the same time, soil temperature at the surface showcases a significant decrease. Meanwhile, the other related factors also tend to show significant variations, such as a decrease in the air temperature during these months. Soil temperature is lowered to 0 °C in phase 4 and reaches to phase level. However, relative humidity is significantly increased during this period. Therefore, Fig. 5 clearly explains the relationship among the variables and highlights the importance of considering these variables to develop the prediction model.

### Correlation analysis among parameters

**Figure 6:**
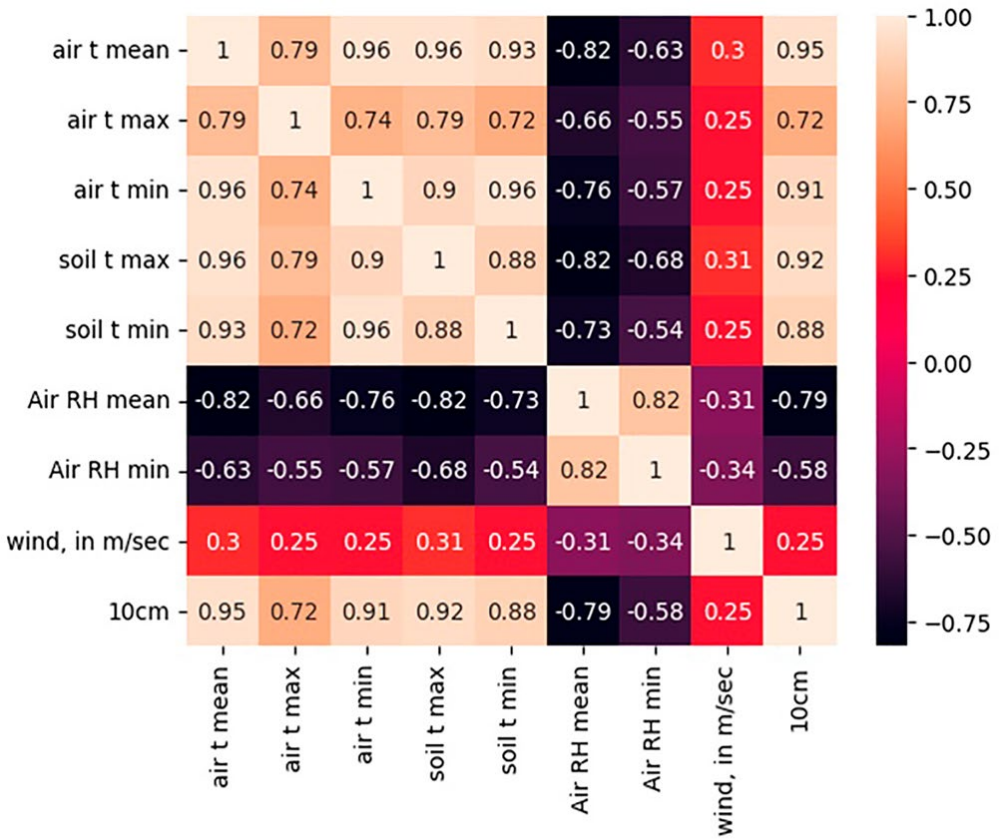
Correlation map for the considered factors for the prediction of the 10 cm depth soil temperature.

Moreover, the Pearson correlation gave the relationships between the considered factors. The results achieved through the correlation test represent the proportionality of the factors. The following Fig. 6 illustrates how the correlation maps lie for the considered factors. It can be clearly seen that soil temperature at 10 cm depth has a good relationship with atmospheric temperature, soil surface temperature, relative humidity, and wind speed. Therefore, all factors were utilized in training the machine learning models.

**Figure 7:**
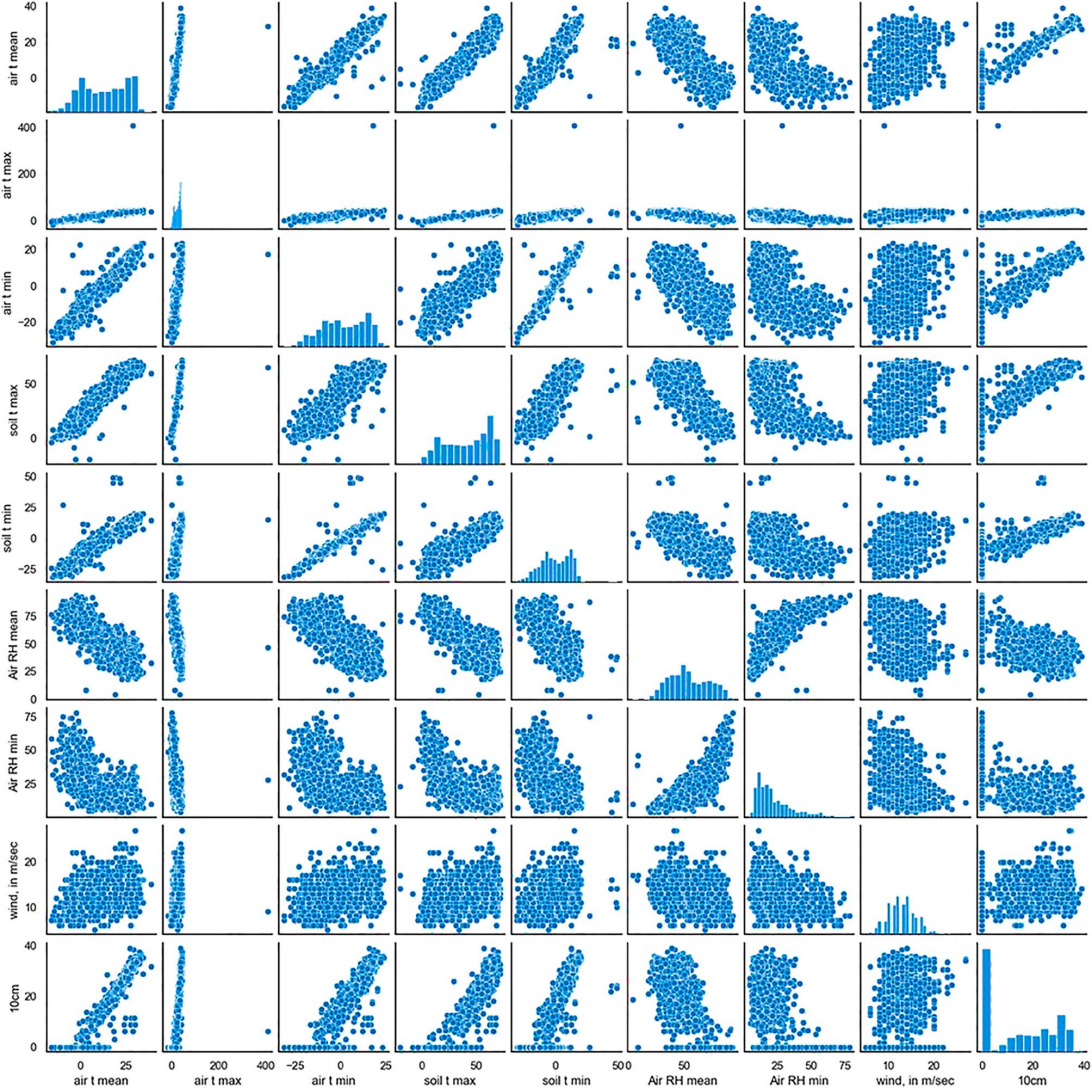
Correlation of the 10 cm depth temperature and other considered factors.

Additionally, the data pattern variations were identified with the scatter plots. Thus, the data-wise correlation was identified for each factor. The following Fig. 7 shows how it varies for different factors.

The negative correlation and the higher positive correlations are well illustrated in Fig. 7. The lowest correlation factor, wind speed, illustrates the plot with a higher amount of scattered data. This presents a weak relationship in-between the 10 cm depth temperature and windspeed. However, the soil temperature at a 10 cm depth is mainly affected by the air temperature and the surface soil temperature. The surface soil layer is directly in contact with the temperature variations of the air. In addition, the surface soil temperature has a direct relation to the 10 cm temperature levels. A Pearson correlation coefficient over 0.9 validates the above point. Even if the Relative Humidity (RH) is negative, the value is higher when considering the values.

### Prediction performance of ML models-soil temperature at surface

**Figure 8:**
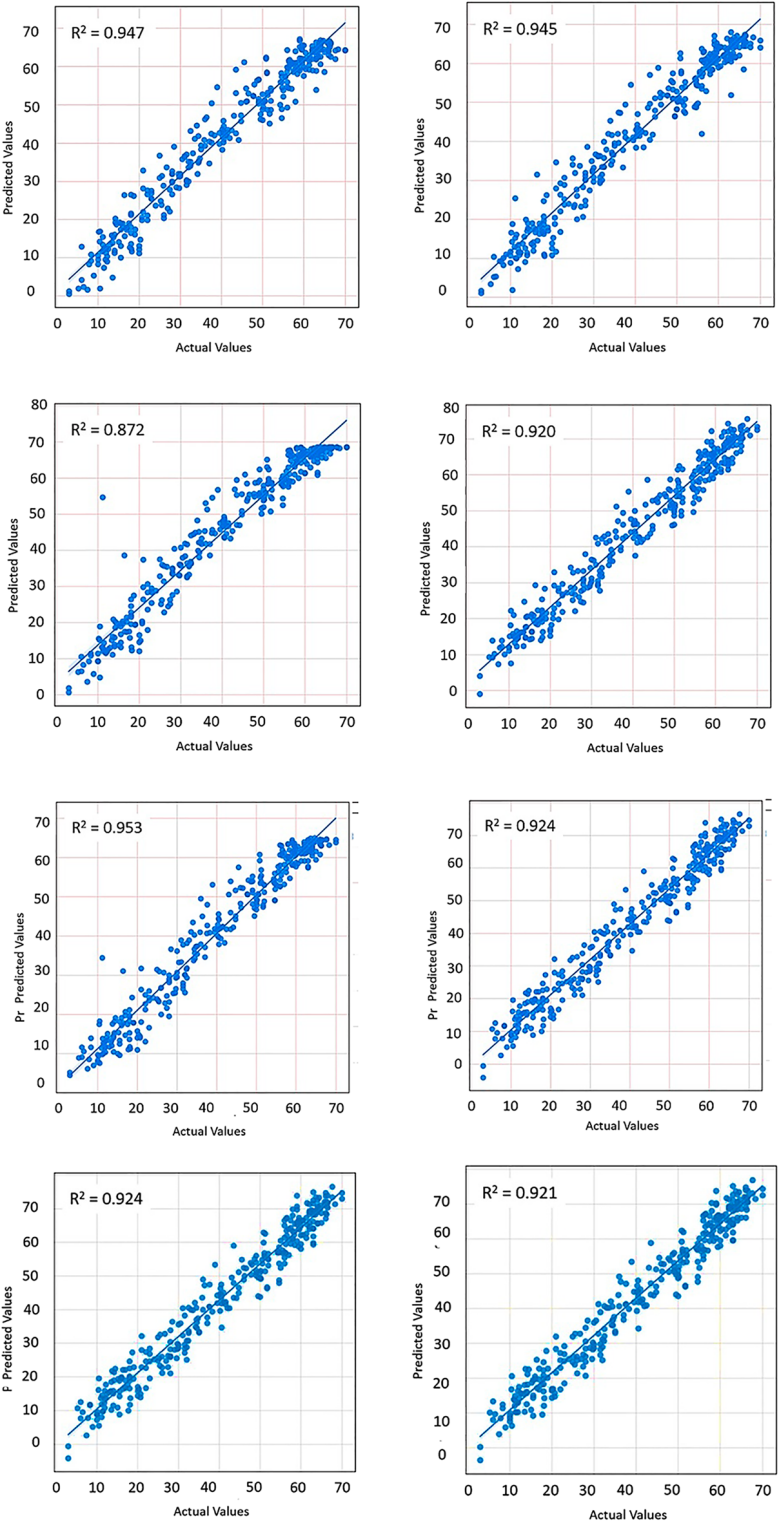
Predicted vs actual—maximum surface temperature at surface.

Figure 8 presents the predicted maximum soil temperature levels against the actual (ground measured) temperature for the soil surface.

Figure 8 presents the scatter view of the coefficient of determination of predicted models. Most of the models perform well on the predictions. Among others, XGBoost can be identified as the best outperforming model for the soil surface maximum value prediction. More details on the prediction performance are given in the Table 2.

**Table 2:** Surface soil temperature maximum value prediction model evaluation.

ML model	R^2^	RMSE	MAE	MSE
XGBoost	0.946	4.398	3.317	19.347
CatBoost	0.944	4.481	3.326	20.084
LSTM	0.871	6.816	5.33	46.472
ANN	0.919	5.398	4.359	29.146
Bi LSTM	0.945	4.129	3.052	17.048
Ridge regression	0.923	5.254	4.282	27.609
Lasso regression	0.923	5.253	4.281	27.599
ElasticNet	0.921	5.349	4.376	28.614

Similarly, Table 3 showcases the performance of the developed models for the prediction of minimum soil temperature levels at the surface. All models perform equally; however, LSTM has a slight edge.

**Table 3:** Surface soil temperature minimum value prediction model evaluation.

ML model	R^2^	RMSE	MAE	MSE
XGBoost	0.811	5.579	2.042	31.136
CatBoost	0.811	5.572	1.996	31.057
LSTM	0.814	5.538	2.013	30.67
ANN	0.798	5.775	2.154	33.356
Bi LSTM	0.806	5.648	2.637	31.904
Ridge regression	0.796	5.796	1.979	33.594
Lasso regression	0.796	5.795	1.990	33.591
ElasticNet	0.798	5.772	1.985	33.316

### Prediction performance of ML models—soil temperature at 10 cm depth

**Figure 9:**
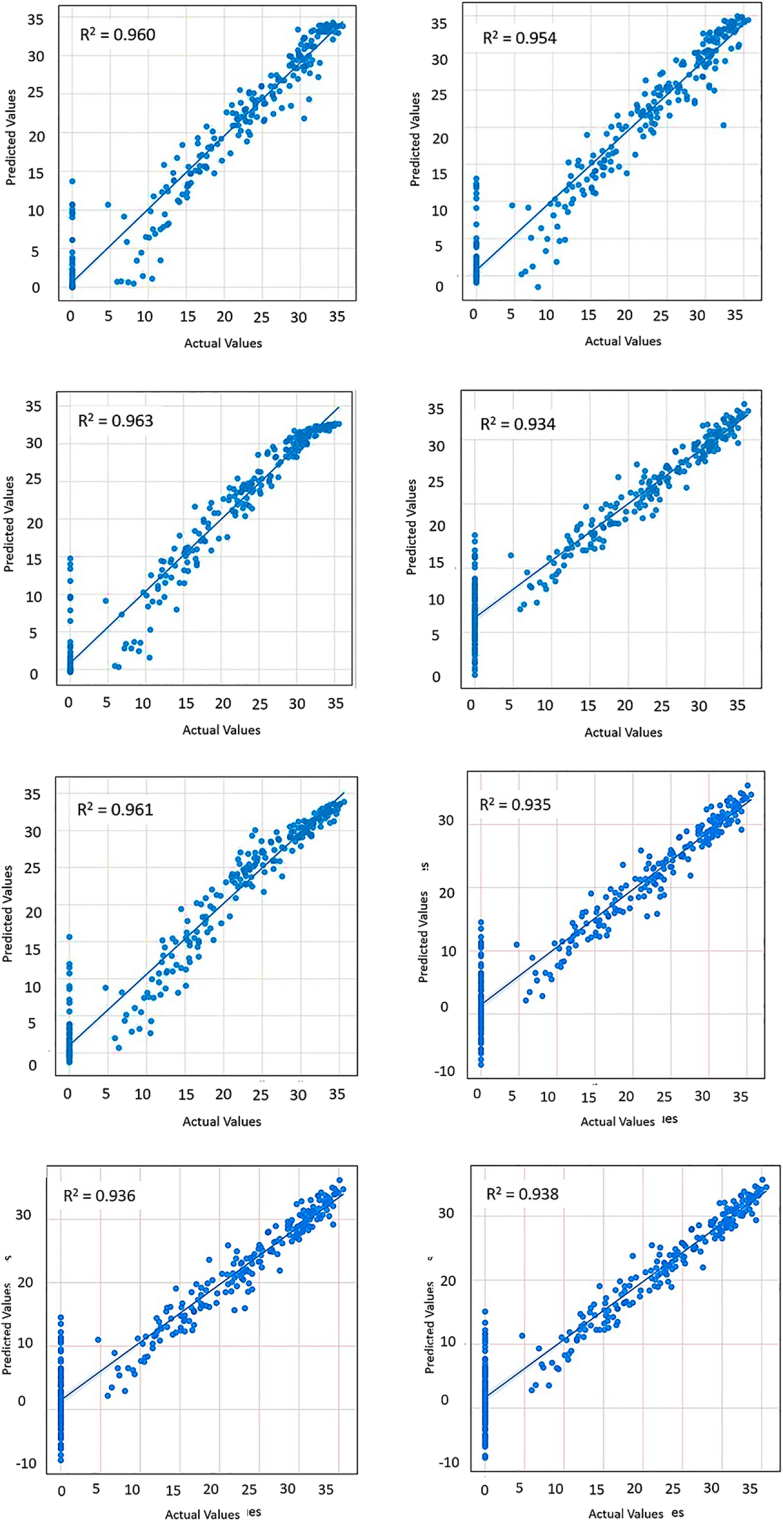
Predicted vs actual—maximum soil temperature at 10 cm depth.

The performance of soil temperature level prediction at 10 cm depth is given in Fig. 9. LSTM is outperforming the remaining models in predicting the 10 cm depth soil temperature. That implies that LSTM can understand the data patterns in the fed dataset. Model performance with respect to other indices is given in Table 4.

**Table 4:** 10 cm depth soil temperature value prediction model evaluation.

ML model	R^2^	RMSE	MAE	MSE
XGBoost	0.959	2.628	1.537	6.907
CatBoost	0.954	2.8	1.688	7.844
LSTM	0.963	2.512	1.433	6.315
ANN	0.934	3.353	2.328	11.245
Bi LSTM	0.961	2.571	1.663	6.614
Ridge regression	0.935	3.326	2.38	11.067
Lasso regression	0.935	3.322	2.376	11.041
ElasticNet	0.937	3.262	2.303	10.646

The performance of all models in prediction is acceptable as per the results shown in Table 4. However, LSTM has the best performance with slight improvement (R^2^ = 0.963; RMSE = 2.512).

### Soil temperature prediction results

**Figure 10:**
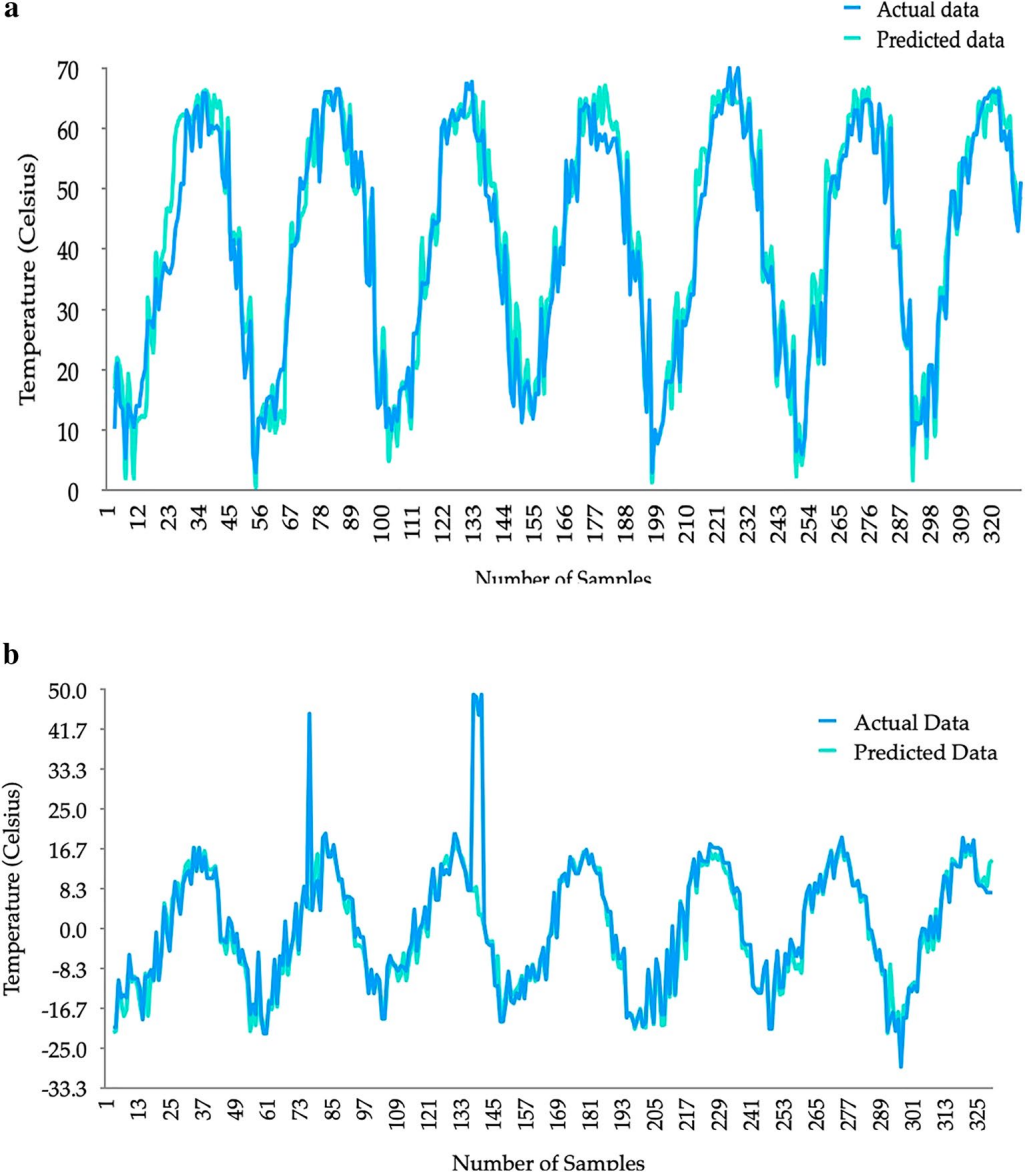
Predicted soil temperature levels at surface.

Figure 10 exhibits the soil temperature predictions for the maximum and minimum temperatures at the surface from the best models. It can be clearly seen that the peaks and troughs were well predicted by XGBoost for the maximum temperature predictions (refer to Fig. 10a). However, two peaks were not captured by LSTM in predicting the minimum soil surface temperature levels. When compared to the other ground-measured temperature levels, these two peaks are abnormal. This could be well due to some sensor issue in the measurements. Nevertheless, all other peaks and troughs in minimum soil temperature levels were predicted accurately (refer to Fig. 10b). Therefore, accurate predictions for both minimum and maximum soil temperature levels at the surface are set by the developed models.

**Figure 11:**
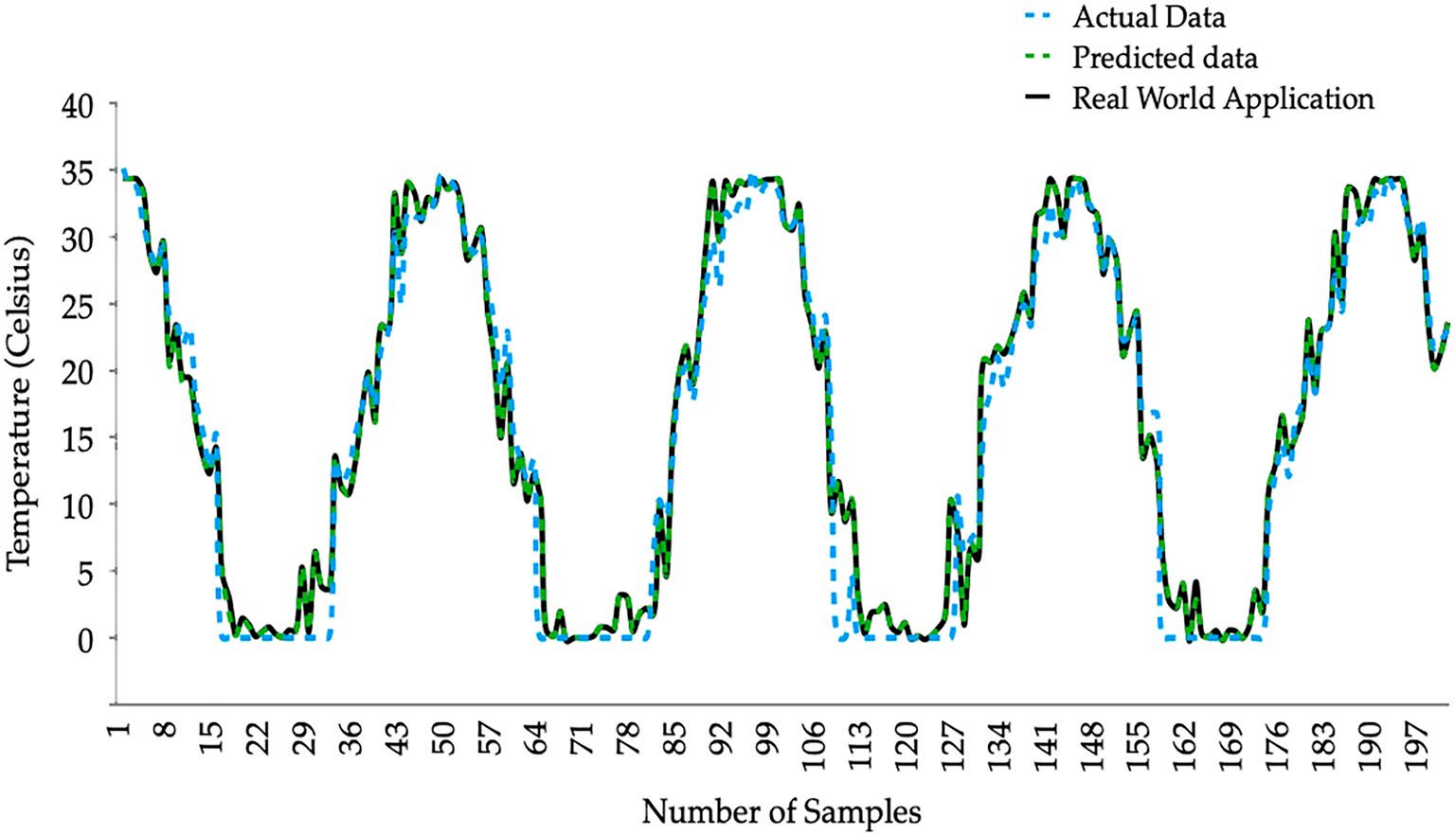
Soil temperature predictions at 10 cm depth.

Figure 11 presents the predicted soil temperature levels at 10 cm depth. The predictions were compared against the measured temperatures. Actual data represents the measured temperature at 10 cm depth. Predicted data is the prediction based on measured surface temperature levels and measured climatic data. However, interestingly as the final outcome of this prediction model, we have predicted the 10 cm depth soil temperature levels using the measured climatic data and predicted soil surface temperature levels which is given as the Real World Application in Fig. 11.

Predictions are smoothly overlapped with the actual temperatures highlighting peaks and troughs. In addition, the seasonal variations are well established. Therefore, the prediction model based on the external climatic data and predicted soil surface temperature levels can be efficiently used to understand the soil temperature levels at 10 cm depth. Thus, the continuous measurements can be minimized in Nukus, Uzbekistan.

In addition, the predicted soil temperatures can be used for sustainable agricultural purposes in arid areas. Chaturanika et al.^48^. Comprehensively presented the importance of water-saving techniques in agriculture for Uzbekistan with the help of mulching agriculture. Soil temperature levels have a direct relationship with mulching agriculture^49^. Therefore, the prediction of soil temperature would encourage the requirement of planning for mulching. This strategy is highly important for a country like Uzbekistan where the water resources are limited. As it was discussed in the introduction, plant growth is directly related to soil temperature levels. With the findings of this research, plant growth can be accurately predicted by amending the model. Not only the plant growth but also the germination is highly impacted by the soil temperature levels^50^. Therefore, the findings are useful in the proper planning of initial processes of agriculture.

Evaporation is another aspect that is directly linked to the soil temperature^51^. The water cycle is unbalanced due to many anthropogenic activities. Therefore, understanding the links to soil temperature under the changing climates would be essential in identifying any possible mitigation strategies for climate change. The soil temperature prediction models have a high role in many aspects to the way of sustainability. Biazar et al.^32^ have developed a soil temperature forecasting model using a hybrid neural network for Florida grazinglands agro-systems. They were able to forecast the soil temperatures to 5, 10, 20, and 50 cm depth and showcased the importance of such a model for agricultural development. Similar findings were showcased by Li et al.^52^ in Qinghai-Tibet Plateau. Therefore, the potential of developing localized models to predict soil temperature levels is highly important in addressing the local issues related to agriculture.

However, the model developed in this research is based on the data from one station at one depth. Therefore, the spatial distribution has not been considered for this analysis. This is due to the lack of reliable data. Therefore, the model can further be developed with more data from nearby areas into a holistic prediction model if data are available. That could lead to developing soil temperature contours and then to understand the impact of these soil temperature contours in agriculture. In addition, the model could have been developed to different depths with more data to cater to the rootzones of the frequent cash crops available in the region. However, the data scarcities have limited the scope of the work.

Nevertheless, the findings of the research are helpful in achieving several Sustainable Development Goals (SDGs). SDG-2; Zero hunger is directly related to the outcome of the research work. In addition, the findings are indirectly helpful in achieving SDG-1; No poverty, SDG-8; Decent work and economic growth, and SDG-13; Climate action. Therefore, the developed model has a greater potential in reaching SDGs. Climate change has made a significant impact in many important areas including water resources, energy production, food production, etc^53,54^. Therefore, achieving SDGs is at the highest challenge.

## Conclusions

Accurate models were developed to predict soil temperature levels at both surface and 10 cm depth for Nukus, Uzbekistan. State-of-the-art machine learning algorithms were used in developing the prediction models. The model developed to predict temperature levels at 10 cm depth is capable of using climatic parameters and predicted soil surface temperature levels as inputs. Therefore, measuring soil surface temperature is not needed to understand the soil behaviour at 10 cm depth. This showcases the greater novelty of the research applied here. However, time-to-time measurements are encouraged to validate the model from time to time under changing climatic conditions. The research presented here can be used to improve the sustainability aspects of food production in arid areas like Nukus, and Uzbekistan. In addition, similar models can be developed for the whole country and understand the soil behaviour. The projected results can be used to develop a countrywide sustainability model not only to look at food production but also to improve biodiversity. Therefore, the findings of this research lead to achieving success in several Sustainable Development Goals. Based on the findings of the research work presented, the following recommendations can be illustrated.

- Soil temperature at 10 cm depth has to be interlinked with the agricultural specialist to understand the best crop types.
- The crop types have to technologically enhanced to climate resilience.
- Frequent monitoring is required to validate the temporal variation of the prediction model with changing climates.
- More spatial data have to incorporated in a holistic prediction model.

## Data Availability

Data used in this paper are available on request for research purposes.
